# Male-killing *Wolbachia* and mitochondrial selective sweep in a migratory African insect

**DOI:** 10.1186/1471-2148-12-204

**Published:** 2012-10-15

**Authors:** Robert I Graham, Kenneth Wilson

**Affiliations:** 1 Lancaster Environment Centre, Lancaster University, Lancaster, LA1 4YQ, UK; 2 School of Biological Sciences, Heydon-Laurence Building A08, University of Sydney, Sydney, NSW, 2006, Australia

**Keywords:** Wolbachia, Cytochrome Oxidase I, COI, mtDNA, Spodoptera exempta, African armyworm, Evolutionary genetics

## Abstract

**Background:**

Numerous recent studies have shown that resident symbiotic microorganisms of insects play a fundamental role in host ecology and evolution. The lepidopteran pest, African armyworm (*Spodoptera exempta*), is a highly migratory and destructive species found throughout sub-Saharan Africa, that can experience eruptive outbreaks within the space of a single generation, making predicting population dynamics and pest control forecasting extremely difficult. Three strains of *Wolbachia* have recently been identified infecting this species in populations sampled from Tanzania. In this study, we examined the interaction between *Wolbachia pipiensis* infections and the co-inherited marker, mtDNA, within populations of armyworm, as a means to investigate the population biology and evolutionary history of *Wolbachia* and its host.

**Results:**

A *Wolbachia*-infected isofemale line was established in the laboratory. Phenotypic studies confirmed the strain *wExe1* as a male-killer. Partial sequencing of the mitochondrial *COI* gene from 164 individual field-collected armyworm of known infection status revealed 17 different haplotypes. There was a strong association between *Wolbachia* infection status and mtDNA haplotype, with a single dominant haplotype, *haplo1* (90.2% prevalence), harbouring the endosymbiont. All three *Wolbachia* strains were associated with this haplotype. This indicates that *Wolbachia* may be driving a selective sweep on armyworm haplotype diversity. Despite very strong biological and molecular evidence that the samples represent a single species (including from nuclear 28S gene markers), the 17 haplotypes did not fall into a monophyletic clade within the *Spodoptera* genus; with six haplotypes (2 each from 3 geographically separate populations) differing by >11% in their nucleotide sequence to the other eleven.

**Conclusions:**

This study suggests that three strains of *Wolbachia* may be driving a selective sweep on armyworm haplotype diversity, and that based on *COI* sequence data, *S. exempta* is not a monophyletic group within the *Spodoptera* genus. This has clear implications for the use of mtDNA as neutral genetic markers in insects, and also demonstrates the impact of *Wolbachia* infections on host evolutionary genetics.

## Background

Recent studies into resident heritable symbiotic microorganisms have highlighted the central role they play in their insect host’s ecology and evolution [1,2]. These symbionts can be classified as either obligate or facultative for host survival. Facultative symbionts are not essential for host development or reproduction, but their presence can impact upon host dynamics by manipulating host reproduction [3-5], as well as increasing host survival or fecundity [6-10], whereas removal of obligate symbionts results in the death of its host [11,12]. One successful group of such symbiotic microorganisms belongs to the genus *Wolbachia.* These are intracellular, maternally-inherited bacteria belonging to the alpha-Proteobacteria group *Rickettsia*. They are among the most successful genus of bacteria, found in filarial nematodes, crustaceans, arachnids, and are estimated to occur in 20 - 70% of all insect species [13,14].

*Wolbachia* have the ability to induce a number of reproductive manipulations of their hosts, such as feminisation of genetic males, induction of sperm-egg incompatibilities, thelytokous parthenogenesis, and male-killing [1,15]. Through these processes, *Wolbachia* provides infected host-females with a relative reproductive advantage over uninfected females [16]. Each of these mechanisms increases the symbiont-infection frequency by ensuring that the number of infected daughters produced by an infected female is greater than the average production of daughters per female. A single insect or population may be infected with more than one symbiotic microorganism and different populations may have different infection statuses [17,18].

Both *Wolbachia* and host mitochondria are maternally transmitted and subsequently can be co-inherited by the offspring. The high mutation rate of mitochondrial DNA makes it a valuable evolutionary marker when compared to corresponding nuclear DNA [19]. With this in mind, it also has the advantage of very low recombination, resulting in the whole mtDNA genome having the same genealogical history [20]. It is well documented that symbionts such as *Wolbachia* can impact upon mtDNA diversity within host populations [21-25]. Indeed, the strong linkage between the two co-inherited markers makes them ideal candidates for the investigation of symbiont invasion-history and symbiont impact upon host genetics [21,26,27]. Previous studies have documented the role of *Wolbachia* in driving dramatic changes within host populations, due to the phenotypes induced by the symbionts [28]. It is now widely accepted that endosymbiont screening and analysis should take place before any attempt to explain mtDNA patterns in terms of host ecology and evolution [20,28].

The larval stage of the African armyworm, *Spodoptera exempta* (Lepidoptera: Noctuidae), is one of the most devastating crop pests in Africa, feeding upon many of the staple food crops such as maize, wheat, sorghum, millet, rice and pasture grasses. Most outbreaks occur on the eastern half of sub-Saharan Africa, as far north as Sudan and as far south as South Africa [29]. The adult moths are highly migratory, often flying hundreds of kilometres over consecutive nights [30], with moth movements largely determined by the seasonal progression of the inter-tropical convergence zone (ITCZ). Therefore, it is believed that early-season armyworm outbreaks in central Tanzania essentially act as “source” populations for moths that will subsequently migrate to northern Tanzania and further northwards towards the horn of Africa [29].

A baculovirus, SpexNPV, is known to be present in field populations of armyworms causing larval mortality, which can have significant impact upon host population dynamics during sporadic natural epizootics. In a recent study, we found that armyworm harboured three strains of *Wolbachia*, designated *wExe1*, *wExe2* and *wExe3*[31]. Based on MLST classification, one of the strains, *wExe1*, was 100% identical to ST-125, which is a male-killing phenotype found in the nymphalid butterfly, *Hypolimnas bolina*[32]*.* The other two strains of *Wolbachia* are new sequence-types, assigned ST-222 (*wExe2* – clade B) and ST-223 (*wExe3* – clade A). Interestingly, both laboratory and field data showed that infection with *wExe1* strain increased host susceptibility to a baculovirus, which could have clear implications for the evolutionary history of the host and pathogen [31].

In the current study, our aim was to address the following questions: (i) How robust is the male-killing phenotype and efficiency of *wExe1* vertical transmission? (ii) In the field, is global *Wolbachia* infection associated with particular host mitochondrial genotypes? (iii) Is there evidence of a selective sweep within the host population? To answer these questions, we established laboratory cultures of *wExe1-*infected armyworm to assess the infection phenotype. We obtained partial sequences of *Wolbachia* genes and the mitochondrial cytochrome oxidase I (*COI*) gene from field-collected *S. exempta* larvae and adults collected from pheromone traps. We examined the diversity of mitochondrial haplotypes, and analysed mtDNA variation to explore the potential association with different *Wolbachia* infection statuses.

## Results

### Phenotypic effects of *wolbachia* infection

Two *wExe1*-infected and several uninfected isofemale lines were established from field-collected pupae, and maintained under laboratory conditions for >4 generations. The two *wExe1*-infected lines had female-biased mean sex ratios of 1:0 (females:males) for the first 3 generations, suggesting an efficient transmission of the infection. On average, only 48.4% (n = 7,525) of the eggs hatched in the infected lines, compared to 94.3% (n = 10,366) in uninfected lines, indicating that *Wolbachia* caused the male-killing phenotype at the embryonic life-stage. In generation 4, a single *wExe1*-infected breeding pair produced a sex ratio of 10:3 (Table 1), suggesting that inefficient transmission can occur within this host-endosymbiont complex. PCR analysis of the offspring from this pair indicated a *Wolbachia* infection rate of 86.7% (n = 30) in the females, and 0% (n = 9) in the males. Tetracycline treatment cured the insects of *Wolbachia* infection, and produced sex ratios and egg hatch rates comparable to naturally uninfected isofemale lines (Table 1), demonstrating that *Wolbachia* was the cause of the observed distortions.

**Table 1:** **Sex ratio and hatch ratio of insect lines reared under laboratory conditions, uninfected and infected with*Wolbachia wExe1*strain**

**Generation**	**Infected**				**Uninfected**				**Tetracycline-treated**			
	**Females (n)**	**Males (n)**	**Ratio**	**Egg hatch (%)**	**Female (n)**	**Male (n)**	**Ratio**	**Egg hatch (%)**	**Female (n)**	**Male (n)**	**Ratio**	**Egg hatch (%)**
1	2	0	1.00	47.97	21	17	0.55	96.72	-	-	-	-
2	303	0	1.00	48.65	433	416	0.51	94.71	30	24	0.56	100
3	378	0	1.00	49.26	216	187	0.54	91.39	170	155	0.52	92.11
4	426	3	0.98	47.51	151	139	0.52	94.74	180	170	0.51	92.84

### *Wolbachia* and host mtDNA *COI* gene diversity

**Figure 1:**
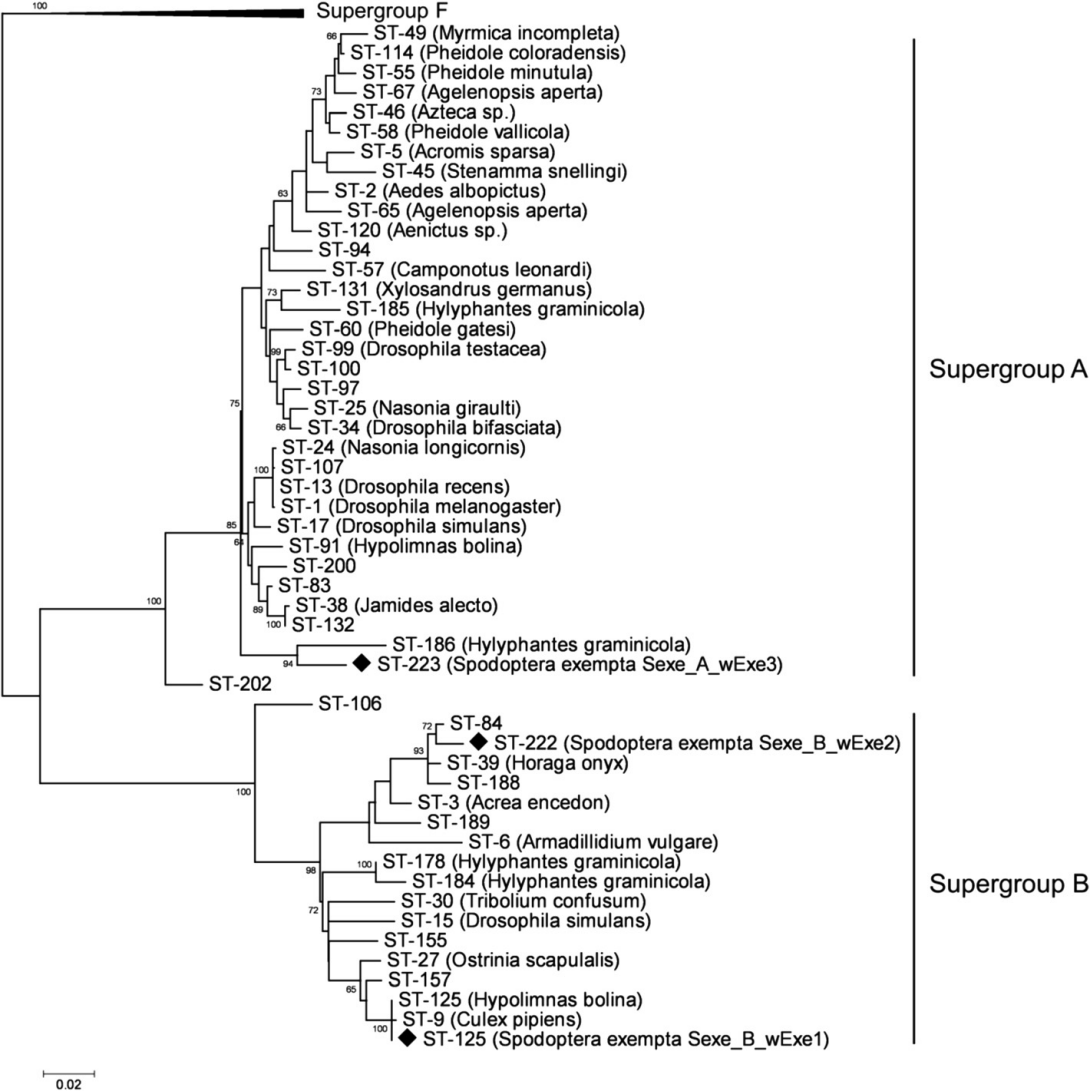
** Maximum-Likelihood (GTR+G+I model) phylogenetic tree for the concatenated MLST genes of *Wolbachia* isolates.** The solid diamonds indicate the 3 strains isolated from *S. exempta* in this study. Where known, the names of the host species are given. The scale bar represents a 2% estimated difference in nucleotide sequence. Numbers given at each node correspond to the percentage bootstrap values (for 1000 repetitions). Replicate numbers of *<*60% were not included in the figure. *Cimex lectularius Wolbachia* ST-8 from Supergroup F is used as an outgroup.

**Figure 2:**
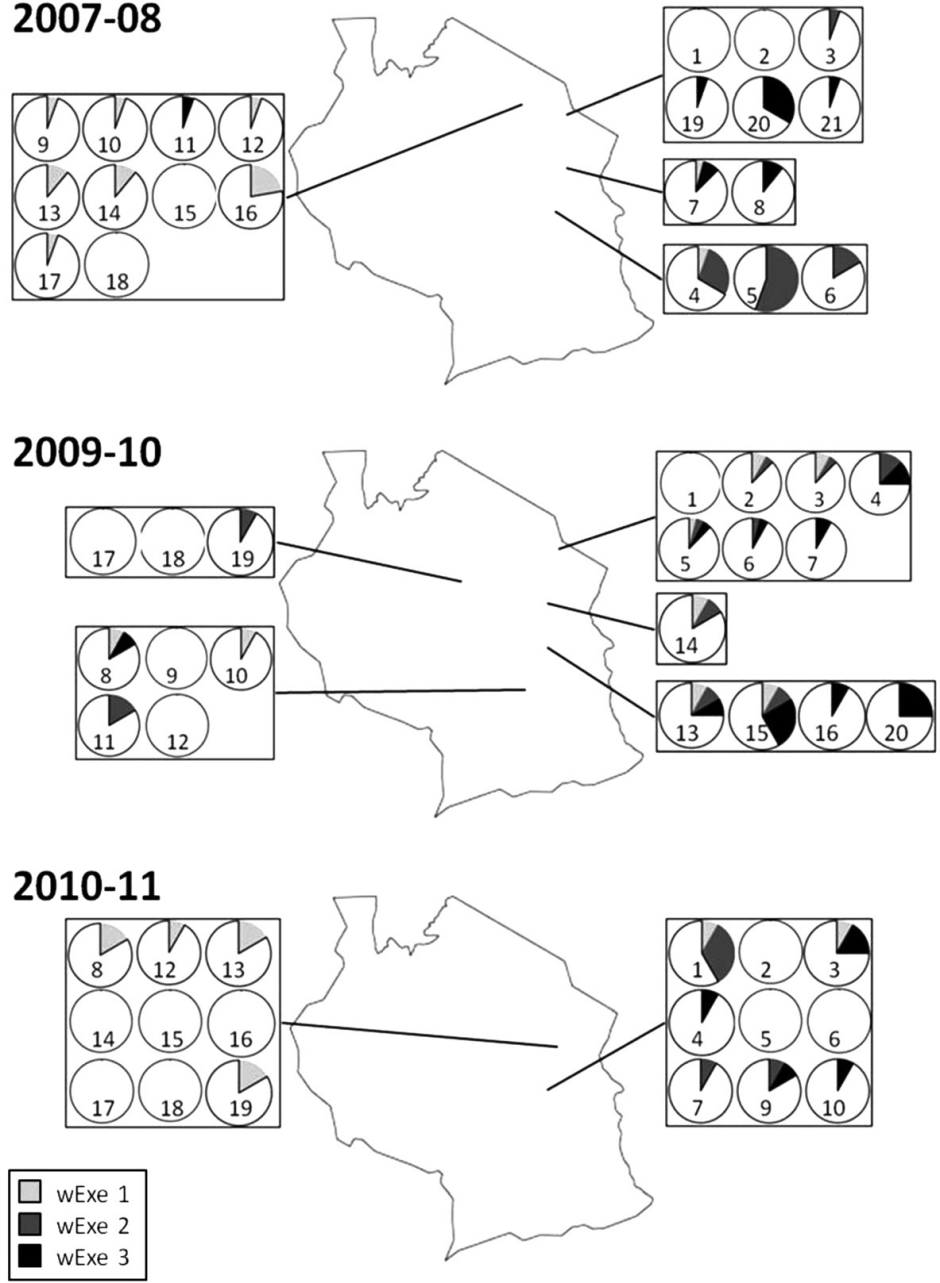
** The spatial prevalence of *Wolbachia* infections within armyworm larval populations sampled throughout Tanzania over the course of three field seasons.** The numbers correspond to the field sites in Additional file 1: Table S1, numbered sequentially through the season.

**Figure 3:**
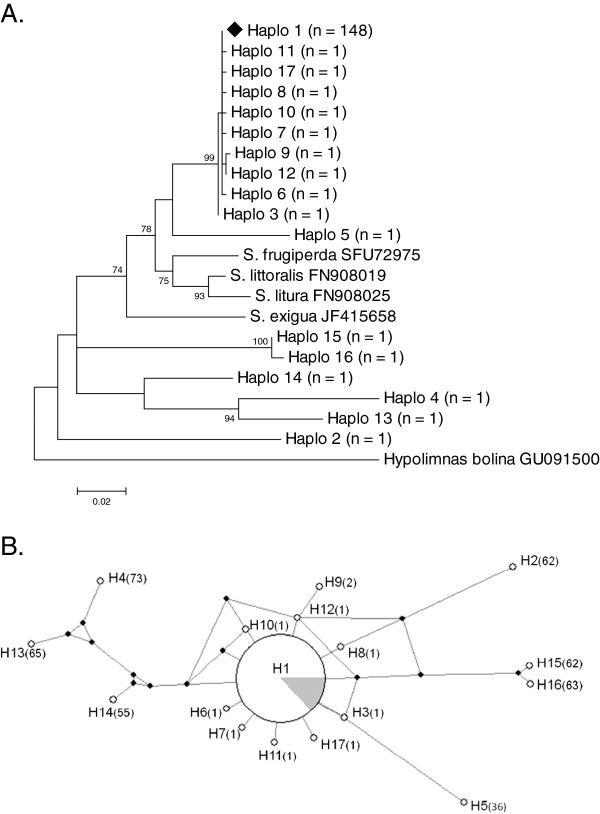
** 3a: Maximum-Likelihood (GTR+R model) phylogenetic tree of the mtDNA COI gene, indicating the paraphyletic nature of the *Spodoptera* genus. ***Haplo1*, the most common of the *S. exempta* haplotypes, is indicated by the solid diamond. The scale bar represents a 2% estimated difference in nucleotide sequence. Numbers given at each node correspond to the percentage bootstrap values (for 1000 repetitions). Replicate numbers of *<*60% were not included in the figure. The nymphalid *Hypolimnas bolina* is used as an outgroup, **3b**: A network analysis displaying the skew and selection for mtDNA haplotype *haplo1* (H1) within the *S. exempta* populations. The filled grey segment in the H1 pie-chart indicates the total percentage of *Wolbachia* infections in the samples, all found within *haplo1* haplotypes. The most divergent haplotypes are found furthest from the centre, and brackets indicate the difference in nucleotide substitutions with *haplo1*. Filled circles indicate “missing” haplotypes in the evolutionary chain.

Larvae (n = 932) were screened for the presence of *Wolbachia* from 59 outbreaks distributed across Tanzania over a 4-year period (see [31]). *Wolbachia* infections were not detected in any of the male moths (n = 334) collected in the 16 pheromone traps (over an area exceeding 750 000 km^2^). Three strains of *Wolbachia* were isolated, with MLST analysis indicating two new strains, and one strain identical to ST-125 (Figure 1; [31]). Strains *wExe1*, *wExe2* and *wExe3* were observed in 23 (39%), 18 (31%) and 19 (32%) of the 59 outbreaks [31], respectively, with 3 outbreaks containing all three strains, 11 with two, 29 with just one, and 16 with no *Wolbachia* present (Figure 2; Additional file 1: Table S1). Overall *Wolbachia* prevalence was 11.9%, 12.0% and 9.3% in sampled larvae over the three outbreak seasons. Mitochondrial COI sequences were obtained from a subsample of larval and adult-males collected from populations throughout Tanzania over two seasons (n = 164). A total of 17 different haplotypes were found in the armyworm samples (Figure 3a) [Genbank: JQ315120 - JQ315136]. The host COI haplotype diversity estimate was found to be low (haplotype diversity, Hd: 0.1861; nucleotide diversity, π: 0.00798). 

**Figure 4:**
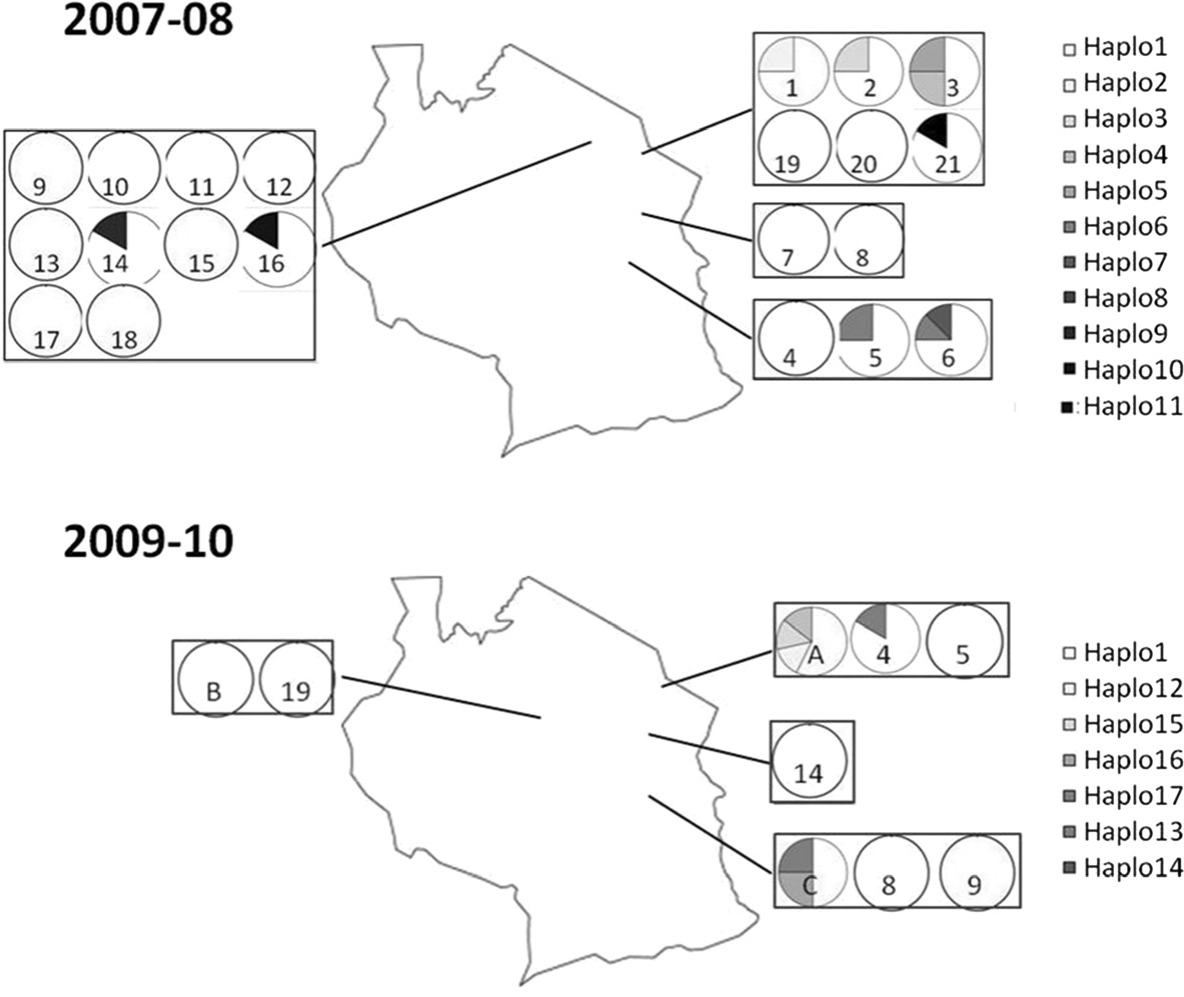
** The spatial prevalence of *COI* hapolotypes within armyworm larval populations sampled throughout Tanzania over the course of two field seasons.** The numbers correspond to the field sites in Additional file 1: Table S1, numbered sequentially through the season. A, B and C refer to the moth trap catches from those districts.

According to neutral evolutionary theory, estimates of Tajima’s *D* and Fu & Li’s D* and F* statistics are expected to equal zero. Positive values are consistent with an excess of intermediate-frequency variants, whereas negative values indicate an excess of rare variants, as can result from a recent population bottleneck or a selective sweep. In the present study, estimates of *D*, D* and F* statistics were all negative for the COI gene (Tajima's *D*: -2.667, p < 0.001; Fu & Li's D*: -3.661, p < 0.02; Fu & Li's F*: -3.824, p < 0.02). Of the 164 sequences obtained, 148 (90.2%) belonged to the same haplotype, assigned *haplo1* (Figure 3b). Significantly, all the *Wolbachia* infections detected in *S. exempta* were found associated with mtDNA *haplo1*, suggesting that recent selective sweeps associated with the invasion of *Wolbachia* have affected mtDNA diversity in the armyworm population, with a skewed prevalence of *haplo1*. Six of the haplotypes did differ by up to 11% in their nucleotide sequence to the other eleven (the largest difference observed was between *haplo1* and *haplo4*; 73 nucleotide substitutions). Eight of the seventeen haplotypes displayed only a single nucleotide difference from *haplo1*, suggesting possible nucleotide substitution events occurring from *haplo1*. Apart from *haplo1*, all of the other haplotypes were very rare, each only detected in a single individual, making any inference on distribution-structuring or migratory behaviour difficult (Figure 4). This is most probably as a result of the highly migratory nature of this host species.

### Host nuclear gene diversity

**Figure 5:**
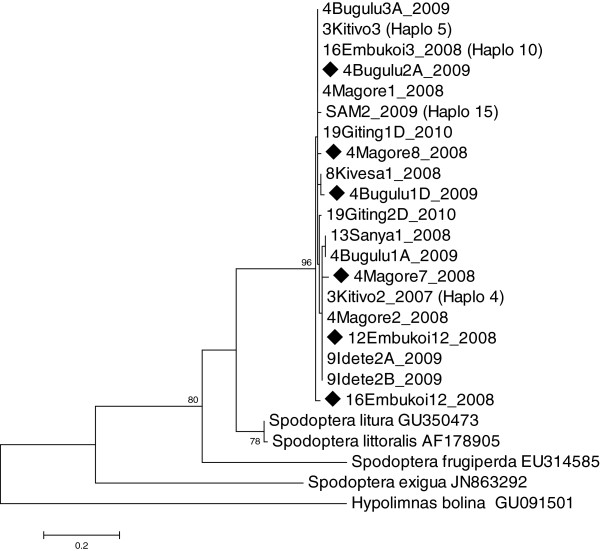
** Maximum-Likelihood (Kimura-2 parameter model) phylogenetic tree of the nuclear DNA 28S gene for armyworm collected at different sites.** The solid diamonds indicate samples that were infected with *Wolbachia*. Samples had H1 haplotypes, unless otherwise indicated. The scale bar represents a 20% estimated difference in nucleotide sequence. Numbers given at each node correspond to the percentage bootstrap values (for 1000 repetitions). Replicate numbers of *<*60% were not included in the figure. The nymphalid *Hypolimnas bolina* is used as an outgroup.

A skew of mtDNA can also result from demographic processes, such as an evolutionary bottleneck, although crucially the latter affects nuclear as well as mitochondrial loci. To investigate this further, we analysed approximately 150 bp of the 28S nuclear ribosomal protein gene from a geographically diverse sub-sample of the insects used to test the haplotypes (n = 20). There was high gene diversity with the vast majority of individuals differing by 1 – 4 nucleotide substitutions (Figure 5; gene diversity: 0.868; nucleotide diversity, π: 0.01400). Estimates of Tajima’s *D* and Fu & Li’s D* and F* statistics were non-significant, suggesting no selection for any particular nuclear genotype (*D*: -1.715, p > 0.05; D*: -1.976, p > 0.10; F*: -2.205, p > 0.05). This suggests that a bottleneck has not occurred, and that the COI skew is a result of a selective sweep, mostly likely driven by the invasion of *Wolbachia* infections.

## Discussion

We explored the evolutionary history of *Wolbachia pipientis* infections in Tanzanian populations of the African armyworm, *S. exempta*. Three strains of *Wolbachia* have recently been detected at an infection prevalence of approximately 12% [31]. Based on previous MLST analysis (using the five genes *ftsZ*, *coxA*, *fbpA*, *hcpA* and *gatB*) [31], one of these strains, *wExe1*, is 100% identical to ST-125, which is a male-killing phenotype found in the Blue Moon Butterfly, *Hypolimnas bolina*[32] and, based on nucleotide identity for *wsp* and *ftsZ* genes, 100% identical to the *Wolbachia* isolate found in Tanzanian populations of the Common Acraea, *Acraea encedon*[33]. This indicates that an infection-transmission event may have occurred between host species at some point in evolutionary history, possibly via a shared generalist parasitoid [34-37]. Indeed, a recent study has shown that plant-mediated horizontal transmission may also be possible in some insect-endosymbiont systems [38]. The phenotypes of strains *wExe2* and *wExe3* are as yet undetermined. However, due to the absence of *Wolbachia* in male adult moths, it is possible that all three strains of *Wolbachia* display the male-killing phenotype. Male-killing *Wolbachia* have been previously identified in both *Wolbachia* Clade A [39] and Clade B e.g. [21,32].

Male-killing is a widespread phenotype in lepidopteran-infecting *Wolbachia* and does appear to explain the sex ratio bias and egg-hatch rate in our infected lab lines. However, we cannot exclude the possibility of a more complicated scenario in the field, as *Wolbachia* is not the only cause of sex ratio distortion to be discovered in insects [40]. Studies on a range of species have revealed a number of insect endosymbionts that can cause similar phenotypic effects to *Wolbachia*[41-43], including the generation of complex fitness interactions between infection-agent and host sex chromosomes, leading to sex-biased mortality [44,45]; DGGE analysis suggests that no secondary endosymbionts occur in the armyworm [31], but this remains an interesting avenue for more detailed future study.

The lack of geographical structuring (of both *Wolbachia* infection status and host mitochondrial genetics) throughout Tanzania is most likely due to the highly migratory nature of the armyworm, thereby preventing genetic differentiations from establishing. This failure to detect any spatial structuring in the armyworm population is consistent with early genetic analyses using iso-enzymes over a much wider East African geographical range [46]. The very large effective population size of this outbreaking species is also likely to be factor. The use of microsatellite markers is currently being examined as a technique allowing more sensitive exploration of the armyworm population structure [47].

Of particular interest was that all three strains of *Wolbachia* were found to be associated with the same host haplotype, *haplo1*. This haplotype was found in 90.2% of samples tested, suggestive of a selective sweep of this haplotype. No other host haplotype was observed to harbour *Wolbachia*, indicative of very low levels of horizontal symbiont transmission between individuals within a population. The high 28S nuclear gene variability, but selection for a single mtDNA haplotype, provides evidence that COI selection is being driven, and that this pattern is not due to demographic processes alone, such as a genetic bottleneck. We propose that this is due to the presence of multiple strains of *Wolbachia* (including the male-killer, *wExe1*). Two hypotheses have been previously proposed for why male-killers reduce mtDNA diversity, causing a sweep [48]. Firstly, the initial invasion of *Wolbachia*, and the subsequent selection for the beneficial parasite genes will result in selective sweeps of the co-inherited mtDNA molecules. Secondly, once the *Wolbachia* invasion has reached equilibrium within the host population, the effective population size of mtDNA will be reduced because mtDNA mutations in uninfected females will tend to be lost [48]. We propose that such a marked *haplo1* sweep has occurred due to three separate *Wolbachia*-strain invasion events associated with mtDNA *haplo1*, each one enhancing the *haplo1* sweep further. It is known that invasion events can cause selection for a particular haplotype, and therefore if three such events occurred in the same haplotype, a haplotype-skew towards 90% prevalence may not be unexpected. However, it must be stated that at this stage of our research, we can only speculate on this possibility.

So why is there such a large haplotype skew (90%) but only a moderate prevalence (12%) of *Wolbachia*? Several hypotheses have been proposed for maintaining *Wolbachia* prevalence equilibrium within host populations, including meta-population structure, mating preference, and environmentally-occurring antibiotics [39,49]. All such spontaneous “cure” events would increase the ratio of uninfected individuals with the same haplotype to infected individuals. We propose two main hypotheses that may explain *Wolbachia* stability in our armyworm system. Firstly, inefficient transmission of male-killing *Wolbachia*. This occurs when an infected female produces offspring that are both infected and uninfected, resulting in individuals of both infection states having the same mtDNA profiles. Results from our laboratory cultures provide evidence for inefficient transmission in the *S. exempta* system, and this phenomenon would certainly help explain the skewed uninfected-*haplo1* distribution. *Wolbachia* infection intensity within an individual may impact greatly upon the transmission efficiency between mother and offspring, whereby a reduction in infection intensity may lead to a decrease in vertical transmission efficiency and consequently loss of infection [50]. In contrast, excessive infection intensity may result in pathology, resulting in negative effects upon host fitness, as seen in the *Drosophilla melanogaster Wolbachia* strain *wMelpop*[51]. A number of previous studies have identified differences in endosymbiont intensity and infection-load within individual hosts [50,52,53]. We are currently developing quantitative assays to assess infection intensity and strain identification for the armyworm-*Wolbachia* system.

Secondly, *Wolbachia*-infected larvae may be more susceptible to an endemic armyworm baculovirus [31]. This would keep a check on *Wolbachia* prevalence in populations by causing greater mortality in *Wolbachia*-infected larvae than non-infected *haplo1* larvae, thereby further exaggerating the *haplo1* uninfected:infected ratio. We propose that a combination of these two mechanisms occurs in the field and contributes to maintaining stability of *Wolbachia* and preserving the mtDNA *haplo1* skew within populations of armyworm. In addition, inter-strain *Wolbachia* stability may exist if the three strains are adapted to different 'environments'. Armyworm experience huge environmental variations over the course of a season e.g. host density, temperature, food-plant changes, disease challenge, etc. The three *Wolbachia* strains may be differentially adapted to hosts experiencing different conditions e.g. those that do better under low or high density conditions, or they interact differently with baculovirus strains. Indeed, in field populations there was no correlation between *wExe3* prevalence and viral mortality, indicating that *wExe3* may not increase virus susceptibility in armyworms (unlike *wExe1* and *wExe2*) [31].

*S. exempta* appears to be paraphyletic at the mtDNA level within the genus *Spodoptera*, with >11% nucleotide divergence amongst haplotypes within what observational and nuclear-gene 28S molecular studies indicate is one species. As previously discussed, mtDNA is a popular method of identifying species, and assessing biodiversity via barcoding protocols [54,55]. Historically these have found intra-specific mtDNA diversity to be very low (typically <1%), and inter-specific diversity to be higher (>2%). However, an increasing number of studies are finding high levels of mtDNA diversity within classical species [25,56], leading to the hypothesis that cryptic species and races are present in greater numbers than previously thought [57,58]. In addition to this, endosymbionts are capable of driving mtDNA introgression from other neighbouring species, thereby confounding the effect of paraphyly within a host species [20,59,60], such as that observed previously in *Drosophila*[61], *Acraea* butterflies [62] and *Stomoxys* flies [63]. This hypothesis may explain the paraphyly patterns observed in our *S. exempta* haplotype phylogeny.

## Conclusion

All three strains of a *Wolbachia* infecting African armyworm *S. exempta* were associated with a single host haplotype, *haplo1*, which comprised 90.2% of the total samples tested. This study suggests that *Wolbachia* is driving a selective sweep for this particular haplotype, and that based on COI diversity, *S. exempta* is not a monophyletic group within the *Spodoptera* genus. This study supports previous research highlighting clear implications for the use of mtDNA as neutral genetic markers in insects.

## Methods

### Collection of armyworm samples and insect rearing

Larval outbreaks were sampled in Tanzania between 2007 and 2011 as previously reported [31]. Briefly, a minimum of thirty live larvae were collected in individual microtubes and stored in 100% ethanol for use in subsequent laboratory analysis. Adult males were caught throughout the season using a network of pheromone traps located across Tanzania. Trained trap operators collected specimens daily, and stored moths in 100% ethanol. The laboratory *Spodoptera exempta* culture was established from larvae collected in central Tanzania in January 2011, and high numbers were maintained at each generation to reduce inbreeding. All larval lines were maintained on a standard wheatgerm-based artificial diet [64] at a constant temperature of 25°C under a 12 hour light/dark cycle. *Wolbachia*-infected and *Wolbachia*-free insect lines were maintained in the same facility to ensure identical breeding conditions.

### *Wolbachia* detection and identification

DNA extractions were performed using the AllPrep DNA/RNA Mini Kit (Qiagen, Crawley, UK) according to the manufacturer's protocol. Larvae were screened for *Wolbachia* infection by PCR amplifying the *Wolbachia*-specific *wsp* and *ftsZ* genes, using the primers *wsp*-81F/*wsp*-691R [65] and *ftsZfl/ftsZrl*[66], respectively. Reaction mixtures (50 μl) contained PCR buffers (10 mM Tris–HCl pH 8.3 at 25°C; 50 mM KCl; 1.5 mM MgCl; 0.001% gelatin), 5 mM each of dATP, dTTP, dCTP and dGTP, 10 mM of the relevant primers, 1 unit Taq polymerase (Qiagen, Crawley, UK) and approximately 10 ng DNA template. PCR was carried out in a Techne TC-512 thermal cycler (Bibby Scientific Ltd., Stone, UK), under the following reaction conditions: (i) 94°C for 5 min, 1 cycle; (ii) 94°C for 30s, 52°C for 30s, 7°C for 30s, 40 cycles; and (iii) 72°C for 5 min, 1 cycle. *Wolbachia* MLST analysis was undertaken using PCR protocols for amplification of the five reported *Wolbachia* MLST genes (*ftsZ*, *coxA*, *fbpA*, *hcpA* and *gatB*) as described elsewhere [35]. PCR reaction-conditions were as above. All PCR products were run on a 1% agarose gel and visualized using ethidium bromide staining. PCR amplicons were purified (PCR Purification Kit, Qiagen), and sequenced (Source Bioscience, UK). The sequence data were analyzed against the *Wolbachia* MLST database (http://pubmlst.org/Wolbachia/).

### Host mtDNA and 28S analysis

To study armyworm haplotype diversity and distribution, amplification of the *S. exempta* mitochondrial cytochrome oxidase I (COI) gene was undertaken, using universal primers LCO-1490 and HCO-1298 [67], which yielded approximately 640bp amplicons. A sample comprising at least four larvae per 2007–08 population (n = 121), and then a random number of larvae and adults from the field season 2009–10 (n = 43) was investigated. Polymerase chain reaction (PCR) mixtures (50 μL) contained PCR buffer (10 mm Tris–HCl pH 8.3 at 25°C, 50 mm KCl, 1.5 mm MgCl, 0.001% gelatin), 10 μm each of dATP, dTTP, dCTP and dGTP, 0.1 μm of each primer, 1 unit *Taq* polymerase (Qiagen, Crawley, UK) and approximately 10 ng genomic DNA template. PCR reactions were as above. All PCR products were run on a 1% agarose gel and visualized using ethidium bromide staining. Nuclear DNA diversity was studied by amplifying a partial segment of the 28S ribosomal gene, using primers *28SFor* 5' AAA GAT CGA ATG GGG AGA TTC ATC 3' and *28SRev* 5' CGT CCT ACT AGG GGA GAA GTG CAC 3' to yield an approximately 150 bp product. PCR reactions were as above. All PCR products were run on a 1% agarose gel and visualized using ethidium bromide staining.

### Assessing *wolbachia* induced phenotype traits

A proportion of all *Wolbachia-*infected armyworm lines used were treated with 0.03% tetracycline (10 mg/ml) to generate uninfected fly lines. This was achieved by feeding the armyworm larval stage, insuring ingestion of sufficient antibiotic to cure the *Wolbachia* infection, as previously described [68]. Following the tetracycline treatment, armyworms were maintained for a generation to recover before experimental use. All tetracycline-treated lines were tested with *Wolbachia*-specific PCR to test for *Wolbachia* infection. Only clean uninfected lines were used in subsequent experiments.

### Statistical and phylogenetic analysis

PCR products were directly sequenced at Source BioScience UK Ltd. (Nottingham, UK). Sequences were viewed using BioEdit [69] and edited to remove universal primer regions. Preliminary identifications against previously published sequences were provided by BLAST [70]. Sequence alignment was performed with ClustalW 1.8 using default parameters [71]. Maximum-Likelihood phylogenetic analysis was performed having selected the most appropriate model based on lowest AIC score (MEGA 5.05 [72]). Sequences obtained in this study were deposited in GenBank [Genbank JQ315120-JQ315136]. Tajima’s *D* test, Fu and Li's D* and F* tests, haplotype diversity and nucleotide diversity were calculated using DnaSP vers. 5.10 [73]. Median-joining haplotype networks were drawn using Network (version 4.6.0.0; [74]).

## Competing interests

Both authors declare that they have no competing interests.

## Authors’ contributions

RG designed the study, produced and analysed the data. KW instigated and supervised the study. Both authors co-wrote and approved the final manuscript.

## Supplementary Material

Additional file 1 **Table S1. ***Wolbachia* infection prevalence within the 59 sampled populations of African armyworm *Spodoptera exempta*. Click here for file
