# Correction: male-killing *Wolbachia* and mitochondrial selective sweep in a migratory African insect

**DOI:** 10.1186/1471-2148-13-6

**Published:** 2013-01-10

**Authors:** Robert I Graham, Kenneth Wilson

**Affiliations:** 1 Lancaster Environment Centre, Lancaster University, Lancaster, LA1 4YQ, UK; 2 School of Biological Sciences, Heydon-Laurence Building A08, University of Sydney, Sydney, NSW 2006, Australia

## Correction

Following publication of this work [1], it was brought to our attention that seven of the mitochondrial COI haplotypes described in this manuscript as *Spodoptera exempta* haplotypes were in fact other species. These have been identified as *Amyna punctum* complex (*haplo2*), *Chrysodeixis acuta* (*haplo4*), *Spodoptera triturata* (*haplo5*), *Vittaplusia vittata* (*haplo13*), *Condica* sp. (*haplo14*) and *Mesogenea varians* (*haplo15* and *haplo16*). As a result, we cannot now support one of our original conclusions suggesting that the *Spodoptera* genus does not appear to be monophyletic. The text describing and discussing this claim in the original manuscript [1] should be disregarded.

However, it should be clearly stated that the main findings of the article, namely that the presence of *Wolbachia* appears to be driving a mitochondrial selective sweep within *S. exempta*, still holds true. Indeed, new analysis strengthens the extent of the skew. Here we present the results of the re-analysis with the corrected data sets along with revisions of the relevant figures.

COI sequences were obtained from 157 *S. exempta* specimens and ten haplotypes identified [Genbank: JQ315120, JQ315122, JQ315125 – JQ315131, JQ315136; Figure 1]; 148 (94.3%) of the haplotypes belonged to *haplo1*. Significantly, all the *Wolbachia* infections detected in *S. exempta* were found associated with mtDNA *haplo1*, suggesting that recent selective sweeps associated with the invasion of *Wolbachia* may have affected mtDNA diversity in the armyworm population. The host COI haplotype diversity estimate was found to be very low (haplotype diversity, Hd: 0.112; nucleotide diversity, π: 0.0002). Estimates of *D*, D* and F* statistics were all negative for the COI gene (Tajima's *D*: -2.157, p < 0.01; Fu & Li's D*: -5.121, p < 0.02; Fu & Li's F*:-4.85017, p < 0.02). Apart from *haplo1*, all of the other haplotypes were very rare, each only detected in a single individual, making any inference on distribution-structuring or migratory behaviour difficult (Figure 2).

**Figure 1:**
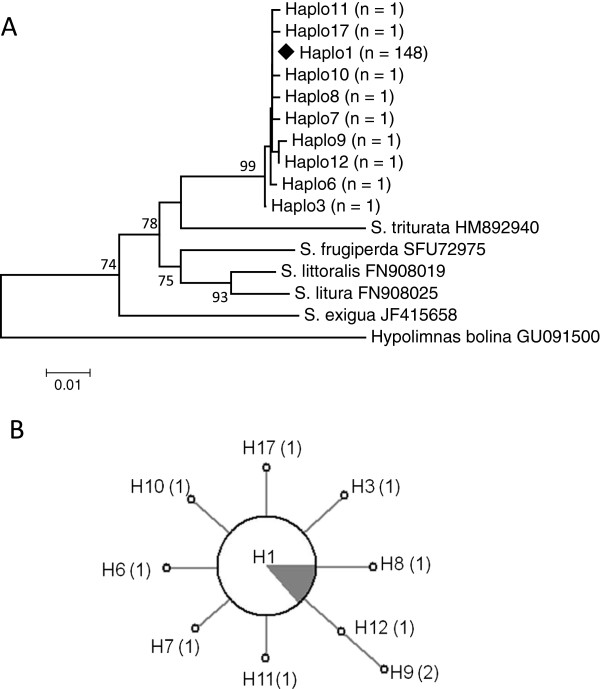
**A: Maximum-Likelihood (GTR+I model) phylogenetic tree of the mtDNA COI gene.***Haplo1*, the most common of the *S. exempta* haplotypes, is indicated by the solid diamond. The scale bar represents a 1% estimated difference in nucleotide sequence. Numbers given at each node correspond to the percentage bootstrap values (for 1000 repetitions). Replicate numbers of *<*60% were not included in the figure. The nymphalid *Hypolimnas bolina* is used as an outgroup. **B:** A network analysis displaying the skew and selection for mtDNA haplotype *haplo1* (H1) within the *S. exempta* populations. The filled grey segment in the H1 pie-chart indicates the total percentage of *Wolbachia* infections in the samples, all found within *haplo1* haplotypes. The most divergent haplotypes are found furthest from the centre, and brackets indicate the difference in nucleotide substitutions with *haplo1*.

**Figure 2:**
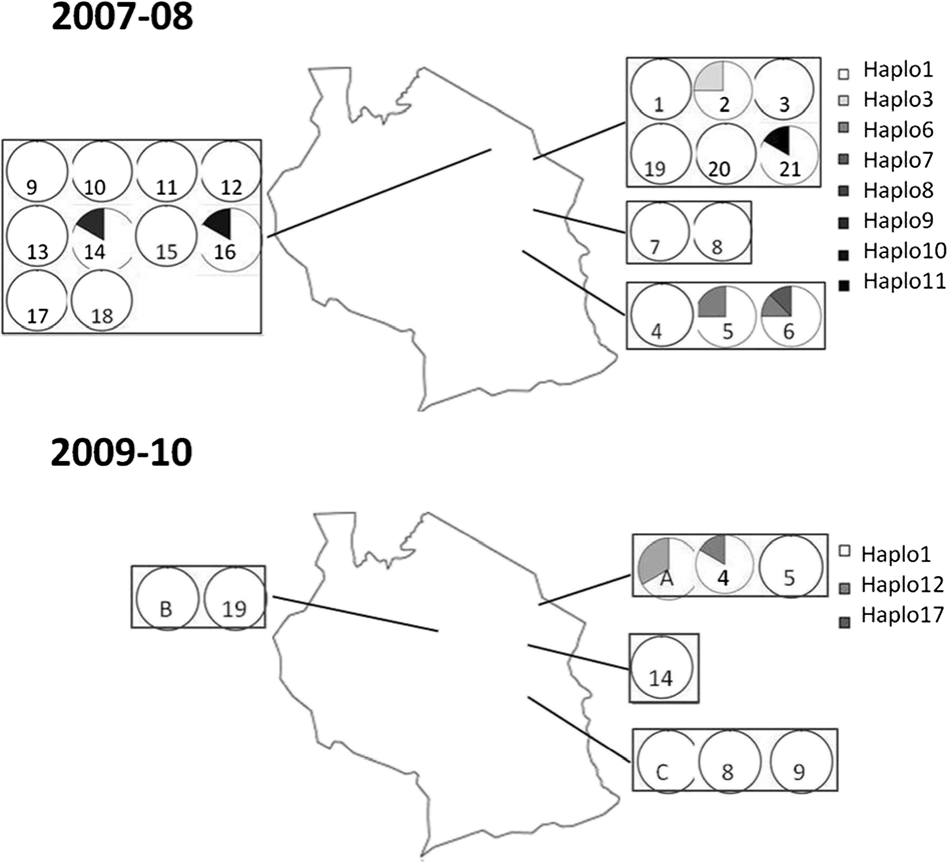
**The spatial prevalence of *COI *hapolotypes within armyworm larval populations sampled throughout Tanzania over the course of two field seasons.** The numbers correspond to the field sites in Table S1, numbered sequentially through the season. **A**, **B** and **C** refer to the moth trap catches from those districts.

We wish to thank Dr. Scott Miller for bringing this matter to our attention.
