# Supplementary material for: Male-killing Wolbachia and mitochondrial selective sweep in a migratory African insect
Source: BMC Evol Biol. 2012 Oct 15;12:204. doi: 10.1186/1471-2148-12-204 (PMC3557208; doi:10.1186/1471-2148-12-204)
Supplement: Additional file 1 — Table S1. Wolbachia infection prevalence within the 59 sampled populations of African armyworm Spodoptera exempta. [file 1471-2148-12-204-S1.pdf]

Supporting Information

Table S1: *Wolbachia* infection prevalence within the 59 sampled populations of African armyworm *Spodoptera exempta*

Season	Site no.	Site	Date collected	no.tested	wExe1	wExe2	wExe3
2007/08	1	Bangala	21/12/2007	16	0	0	0
2007/08	2	Makanya	22/12/2007	18	0	0	0
2007/08	3	Kitivo	23/12/2007	18	0	1	0
2007/08	4	Magole	12/01/2008	18	1	5	0
2007/08	5	Buti Visaraka	13/01/2008	18	0	10	0
2007/08	6	Msimba	14/01/2008	18	0	3	0
2007/08	7	Vibaoni	11/02/2008	24	1	0	2
2007/08	8	Kivesa	12/02/2008	28	0	0	3
2007/08	9	Mungushi	02/04/2008	18	1	0	0
2007/08	10	Mwangaza	02/04/2008	18	1	0	0
2007/08	11	Olumerili	03/04/2008	18	0	0	1
2007/08	12	Embukoi	03/04/2008	18	1	0	0
2007/08	13	Sanya	04/04/2008	18	2	0	0
2007/08	14	Bomani	04/04/2008	18	2	0	0
2007/08	15	Kwa Lodo	05/04/2008	18	0	0	0
2007/08	16	Embukoi	07/04/2008	18	4	0	0
2007/08	17	Lerai	08/04/2008	18	1	0	0
2007/08	18	Geza Ulole	08/04/2008	18	0	0	0
2007/08	19	Ishnde	13/04/2008	18	0	0	1
2007/08	20	Kisima	14/04/2008	18	0	0	6
2007/08	21	Mgagao	14/04/2008	18	0	0	1
2009/10	1	Mwembe V	16/12/2009	24	0	0	0
2009/10	2	Katani	17/12/2009	24	2	1	0
2009/10	3	Katani	17/12/2009	24	2	1	0
2009/10	4	Bugulu	19/12/2009	24	0	3	3
2009/10	5	Katani	19/12/2009	24	1	1	1
2009/10	6	Mgareni	20/12/2009	24	0	1	1
2009/10	7	Katani	21/12/2009	24	0	0	2
2009/10	8	Lumemo	28/12/2009	12	1	0	1
2009/10	9	Idete	29/12/2009	12	0	0	0
2009/10	10	Ihanga	29/12/2009	12	1	0	0
2009/10	11	Kiberege	30/12/2009	12	0	2	0
2009/10	12	Kidatu	31/12/2009	12	0	0	0
2009/10	13	Magole	31/12/2009	12	1	1	1
2009/10	14	Mombo	27/01/2010	12	1	1	0
2009/10	15	Mandela	28/01/2010	12	1	1	3
2009/10	16	Kanzeni	29/01/2010	12	0	0	1
2009/10	17	Loto	03/02/2010	12	0	0	0
2009/10	18	Gocho	04/02/2010	12	0	0	0
2009/10	19	Giting	04/02/2010	12	0	1	0
2009/10	20	Morogoro	29/01/2010	12	0	0	3
2010/11	1	Chanzuru	10/01/2011	12	1	4	0
2010/11	2	Msimba	11/01/2011	12	0	0	0
2010/11	3	Teko	12/01/2011	12	1	0	2
2010/11	4	Chanzuru	12/01/2011	12	0	0	1
2010/11	5	Mbigiri	22/01/2011	12	0	0	0
2010/11	6	Msufini	22/01/2011	12	0	0	0
2010/11	7	Msufini	24/01/2011	12	0	1	0
2010/11	8	Kwemazandu	26/02/2011	12	2	0	0
2010/11	9	Mandela	28/02/2011	12	0	1	1
2010/11	10	Makuyu	01/03/2011	12	0	0	1
2010/11	11	Magole	01/03/2011	-	-	-	-
2010/11	12	Kweingoma	08/04/2011	12	1	0	0
2010/11	13	Mgambo	08/04/2011	12	2	0	0
2010/11	14	Mkokola	09/04/2011	12	0	0	0
2010/11	15	Emau	09/04/2011	12	0	0	0
2010/11	16	Mtonga	10/04/2011	12	0	0	0
2010/11	17	Mandela	10/04/2011	12	0	0	0
2010/11	18	Kwengoma	11/04/2011	12	0	0	0
2010/11	19	Bagamoyo	11/04/2011	12	2	0	0
